# Metabolomic Profiling of Second-Trimester Amniotic Fluid for Predicting Preterm Delivery: Insights from NMR Analysis

**DOI:** 10.3390/metabo13111147

**Published:** 2023-11-12

**Authors:** Charalampos Kolvatzis, Paris Christodoulou, Ioannis Kalogiannidis, Konstantinos Tsiantas, Ioannis Tsakiridis, Charikleia Kyrkou, Antigoni Cheilari, Nikolaos S. Thomaidis, Panagiotis Zoumpoulakis, Apostolos Athanasiadis, Alexandra-Maria Michaelidou

**Affiliations:** 1 Third Department of Obstetrics and Gynecology, School of Medicine, Faculty of Health Sciences, Aristotle University of Thessaloniki, 54642 Thessaloniki, Greece; ikalogia@auth.gr (I.K.); igtsakir@auth.gr (I.T.); apathana@auth.gr (A.A.); 2 Department of Food Science and Technology, University of West Attica, Ag. Spyridonos, 12243 Egaleo, Greece; pchristodoulou@uniwa.gr (P.C.); ktsiantas@uniwa.gr (K.T.); pzoump@uniwa.gr (P.Z.); 3 Department of Food Science and Technology, Faculty of Agriculture, Forestry and Natural Environment, School of Agriculture, Aristotle University of Thessaloniki, 54124 Thessaloniki, Greece; kyrkoucg@agro.auth.gr (C.K.); amichail@agro.auth.gr (A.-M.M.); 4 Department of Pharmacognosy and Natural Products Chemistry, Faculty of Pharmacy, National and Kapodistrian University of Athens, Panepistimiopolis Zografou, 17551 Athens, Greece; cheilarianti@pharm.uoa.gr; 5 Laboratory of Analytical Chemistry, Department of Chemistry, National and Kapodistrian University of Athens, Panepistimiopolis Zografou, 15771 Athens, Greece; ntho@chem.uoa.gr

**Keywords:** amniotic fluid, preterm delivery, NMR metabolomics, multivariate analysis

## Abstract

Preterm delivery (PTD) is a notable pregnancy complication, affecting one out of every ten births. This study set out to investigate whether analyzing the metabolic composition of amniotic fluid (AF) collected from pregnant women during the second trimester of pregnancy could offer valuable insights into prematurity. The research employed ^1^H–NMR metabolomics to examine AF samples obtained from 17 women who gave birth prematurely (between 29^+0^ and 36^+5^ weeks of gestation) and 43 women who delivered at full term. The application of multivariate analysis revealed metabolites (dimethylglycine, glucose, myo-inositol, and succinate) that can serve as possible biomarkers for the prognosis and early diagnosis of preterm delivery. Additionally, pathway analysis unveiled the most critical metabolic pathways relevant to our research hypothesis. In summary, these findings suggest that the metabolic composition of AF in the second trimester can be a potential indicator for identifying biomarkers associated with the risk of PTD.

## 1. Introduction

Spontaneous preterm delivery (PTD), affecting one out of every ten births, is recognized as a syndrome influenced by multiple contributing factors [1]. Among the spectrum of suspected causes of PTD, infection and/or inflammation characterized as the body’s response to signals of microbial or non-microbial danger stand out as the only pathological processes for which a confirmed causal connection with PTD has been established, along with a clearly defined molecular pathophysiology [2].

Infants born prematurely, particularly those born before 34 weeks of gestation, have an elevated risk of mortality and health problems. Furthermore, infants born during the late preterm period, i.e., 34–37 weeks of gestation, face increased health complications and a higher probability of developing health conditions like obesity, metabolic syndrome, hypertension, and type 2 diabetes later in life [3].

Prediction and early diagnosis of PTD are often challenging because of their complexity [4]. Hence, it is not surprising that metabolomics, utilizing advanced techniques such as Nuclear Magnetic Resonance Spectroscopy (NMR), Gas Chromatography–Mass Spectrometry (GC-MS), and Liquid Chromatography–Mass Spectrometry (LC-MS/MS), have gained prominence in identifying biomarkers and pathways crucial to the development and progression of this syndrome [4,5,6,7,8,9,10].

Among the various options, amniotic fluid (AF) emerges as a particularly promising biofluid; it serves as a dynamic repository, reflecting the metabolic profile of the developing fetus. Research studies [11,12,13,14,15] suggest that conducting a metabolomic analysis on AF obtained during the prenatal period holds the potential to identify metabolic deviations before PTD occurs. However, a recent study using untargeted LC-MS mid-trimester amniotic fluid metabolic profiling on two groups of 37 pregnant women (full term/preterm) that underwent amniocentesis showed no evidence of metabolite differentiation for spontaneous PTD [16].

Studies in this area have employed diverse metabolomics techniques, and the consensus on critical metabolites serving as biomarkers remains elusive [10]. The apparent inconsistency highlights the intricate nature of the PTD syndrome and implies the presence of unexplored aspects. Within this context, our study aims to bridge this gap by identifying potential predictive biomarkers for spontaneous PTD within second-trimester AF using NMR analysis.

## 2. Materials and Methods

### 2.1. Study Design and Population

This prospective cohort study investigated AF samples between 2013 and 2014 at the Third Department of Obstetrics and Gynecology, School of Medicine, Faculty of Health Sciences, Aristotle University of Thessaloniki, Greece. The study was approved by the Aristotle University of Thessaloniki Research Ethics Committee (Prot. No. 1.662/21 November 2018) and conducted in compliance with the declaration of Helsinki. No incentives were provided, and all the participants signed a relevant informed written consent.

#### Data Collection and Eligibility Criteria

All the pregnant women in singleton pregnancies who underwent amniocentesis (between 16 and 22 weeks of gestation) for prenatal screening were eligible to participate in the study; a small amount of AF was donated and kept at −80 °C. Detailed obstetric and medical histories were recorded for each woman in the study.

Exclusion criteria were (1) multiple pregnancies, (2) short cervical length at second-trimester ultrasound (<25 mm), and (3) preterm prelabor rupture of membranes, placental ischemic disease, and iatrogenic PTD. In addition, pregnant women who had a miscarriage before 24 weeks or PTD within the first 3 weeks of amniocentesis were excluded from the analysis.

With regards to the indications of amniocentesis, increased risk of fetal genetic abnormality after combined first-trimester ultrasound and biochemical screening, detection of ultrasound markers at first- and second-trimester screenings associated with an increased likelihood of chromosomal abnormalities (e.g., nasal bone hypoplasia, short femur, and hyperechoic bowel), maternal desire, and high risk of vertical transmission of congenital infection were the most common ones, as recommended by the majority of the guidelines [17].

Pregnancy outcomes were collected to determine the gestational age at delivery (preterm <37 weeks or term >37 weeks), mode of delivery (vaginal/cesarean), birthweight, and possible complications during pregnancy. The metabolomic profile of the AF was checked and compared according to the week of delivery and birthweight.

### 2.2. NMR Metabolomics Analysis

#### 2.2.1. Sample Preparation

Amniotic fluid samples were thawed at room temperature (25 °C) and extracted according to a common method established in the literature [13]. Specifically, 10 mL of AF was centrifuged (14,000 rpm, 4 °C, 10 min), and 1 mL of the supernatants was lyophilized overnight until dry. The dry residues were then reconstituted into 540 µL of phosphate buffer (0.2 M, Na_2_HPO_4_ 2H_2_O, and NaH_2_PO_4_, pH = 7.0) in D_2_O and 60 µL (5 mM) of *d6*- trimethylsilyl propionic acid sodium salt (TSP) as the internal standard.

#### 2.2.2. ^1^H–NMR Analysis

For ^1^H–NMR measurement, 600 μL of the final samples was transferred to 5 mm NMR tubes (LabScape, Bruker, Germany). The NMR spectra were acquired at 300 K, after a 5 min resting period for temperature stabilization, on a Bruker Ascend 500 MHz NMR spectrometer equipped with a 5 mm double resonance broadband inverse (BBI) detection probe. Experiments were performed in automation mode, using a SampleCase-24 sample changer operated by IconNMR. Data acquisition and processing were performed with TopSpin 4.1.4 (Bruker Italia Slr, Milan, Italy). Metabolic profiling 1D NMR spectra were acquired using water suppression. T2-edited Carr-Purcell-Meiboom-Gill (CPMG) experiments were acquired with d1 = 6 s; AQ = 4.92 s; FID data points = 96 k; SW = 20 ppm; ns = 32. The transmitter offset was set manually to achieve optimal suppression of the residual water signal for both experiments. FIDs were zero-filled and multiplied by an exponential weighting function corresponding to a line broadening of 0.3 Hz before Fourier transformation. Chemical shift values were referenced to the residual TSP signal (0.00 ppm).

#### 2.2.3. Data Processing

All ^1^H–NMR spectra were phase-corrected using TopSpin 4.1.4 software. Then, the .zip file of all files was uploaded to the NMRProcFlow open-access web tool [18] to proceed with chemical shift calibration, baseline correction, and S/N ratio identification as well as alignment normalization and bucketing. For alignment, an interactive philosophy was performed, meaning that each interval had been chosen separately performing CluPA, as well as the least squares method [2]. All spectra were normalized using the Probabilistic Quotient Normalization (PQN) method [3], and an intelligent bucketing module was performed within the 0.8–8.5 spectra region.

#### 2.2.4. Metabolites Screening

A series of 2D TOCSY and HSQC experiments were acquired for metabolite identification. More specifically, the above validation NMR experiments were acquired using a phase-sensitive MLEV sequence with d1 = 4 s; FID data points = 2 k (F2) and 256 (F1); SW = 20 ppm; ns = 32; and mixing time (d9) = 0.08 sec with suppression of the residual water signal. Furthermore, phase-sensitive HSQC-DEPT experiments were acquired using Echo/Antiecho-TPPI gradient selection with decoupling during acquisition (hsqcedetgpsisp2.3) with FID data points = 4 k (F2) and 288 (F1); SW = 12 ppm (F2) and 180 (F1); and ns = 160 in non-uniform sampling (NUS) acquisition mode with a NUS level of 50%.. To aid in rapid and efficient metabolite identification, Metabominer [19], an easy-to-use software tool, along with 2D TOCSY and HSQC experiments and data from the literature were used. Briefly, an automated pick-picking list from each spectrum (TOCSY and HSQC) was generated from MestreNOVA software and loaded in the Metabominer tool to (a) screen metabolites from Metabominers’ biofluids database and (b) refine the identified compounds by superimposing the spectral images to the Metabominers’ generated spectra.

### 2.3. Statistical Analysis

#### 2.3.1. Demographics

As all demographic parameters did not follow the normal distribution, the Mann–Whitney U test was used for group comparisons. The statistical significance level was set at 5%. The demographics were statistically analyzed with SPSS (Statistical Package for the Social Sciences) v.26 (SPSS, Inc., Chicago, IL, USA).

#### 2.3.2. Metabolomics

The MetaboAnalyst 5.0 was facilitated for both univariate/multivariate analyses and pathway analysis [5]. For multivariate analysis, -Pareto scaling was used, and unsupervised/supervised models (PCA/PLS-DA) were extracted at a confidence level of 95%. Feature selection of the PLS-DA model was based on variable importance projection (VIP) scores > 1.0 to reveal the variable that mostly contributes to the discrimination of the studied groups. The validation of the models was evaluated in terms of accuracy (>90.0 %), the goodness-of-fit R^2^ (0 ≤ R^2^ ≤ 1), and the predictive ability Q^2^ (0 ≤ Q^2^ ≤ 1) values. The PLS-DA results were further cross-validated by carrying out permutation tests with 1000 random permutations.

In terms of univariate analysis, non-parametric Wilcoxon *t*-tests were performed (*p* < 0.05). For the elucidation of metabolites as possible biomarkers, Receiver Operating Characteristic (ROC) curves were acquired. Finally, pathway analysis was performed by Metaboanalyst 5.0 platform.

## 3. Results

In total, 60 AF samples were used in the analyses; 43 were term and 17 were preterm. The mean duration of gestation was 38.5 (±0.9) weeks for the term group and 35.3 (±2.8) weeks for the preterm group (*p* < 0.001). Similarly, the mean birthweight was statistically different with term neonates having a mean birthweight of 3340 g (±380.77), while the preterm ones weighed 2695 g (±553.85) (*p* < 0.001). All parameters investigated are presented in Table 1.

### 3.1. NMR Analysis

**Figure 1:**
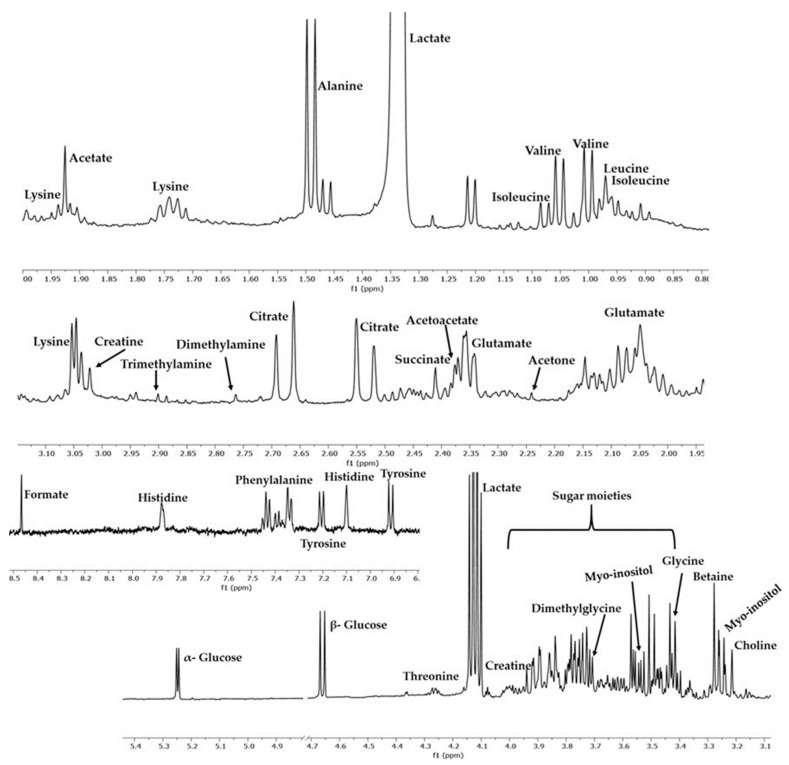
^1^H NMR spectra of AF sample with annotation of the identified metabolites.

A total of 27 metabolites including aliphatic and aromatic amino acids, sugars, and organic acids were assigned by the combination of 2D NMR spectroscopy (2D-HSQC, 2D TOCSY) and available online tools such as Metabominer and the literature data, as described in Section 2.2.4. The identified metabolites of the acquired AF samples are displayed in Figure 1, while the characteristic chemical shifts are represented in Supplementary Table S1.

The spectra matrix underwent intelligent bucketing to reduce the spectra data and facilitate subsequent statistical analysis. A total of 170 buckets were annotated according to the assignment procedure (Supplementary Table S2). The new matrix was subjected to both multivariate and univariate analysis.

### 3.2. Statistics

#### 3.2.1. Overview of the Studied Samples

**Figure 2:**
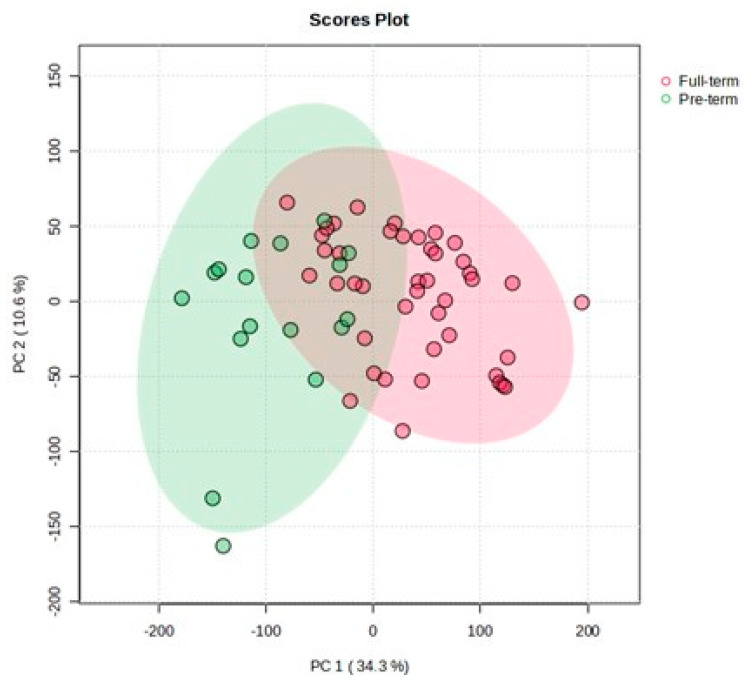
PCA score plot of the studied AF samples; No. of components = 2, *n* = 60, confidence level = 95%; red dots correspond to full-term samples, while green dots correspond to preterm samples.

Principal Component Analysis (PCA) was conducted on the annotated NMR profile (170 annotated intelligent buckets, Supplementary Table S2) to gain an overview of the sample space. The resulting scores plot (Figure 2) revealed a pattern between the two studied groups along the first principal component, accounting for 34.3% of the metabolic variance in the studied AF samples.

#### 3.2.2. Data Reduction Method for Unique Potential Biomarker Discovery

Digging deeper into the annotated metabolites (Supplementary Table S2), it is observed that a significant number of buckets correspond to more than one metabolite. Considering that the purpose of this study is the development of reliable potential prognostic markers for PTD, we applied a robust statistical methodology based on the annotated spectra buckets.

In detail, a gradual bucketing reduction method was implemented by including buckets that characterize unique metabolites to avoid the metabolites’ overlapping phenomenon. This procedure led to the reduction of the feature space to 94 out of 170 annotated intelligent buckets. Notably, the 94 buckets corresponded to the initial number of assigned metabolites (*n* = 27).

Moreover, the reduced feature space (*n* = 94) was subjected to biomarker analysis considering the area under the ROC curve (AUROC) and the *p* values promoting potential biomarkers with a good predictive ability and strong statistical significance (AUROC > 0.75 and *p* < 0.05). These values are in accordance with the literature [10,20].

**Figure 3:**
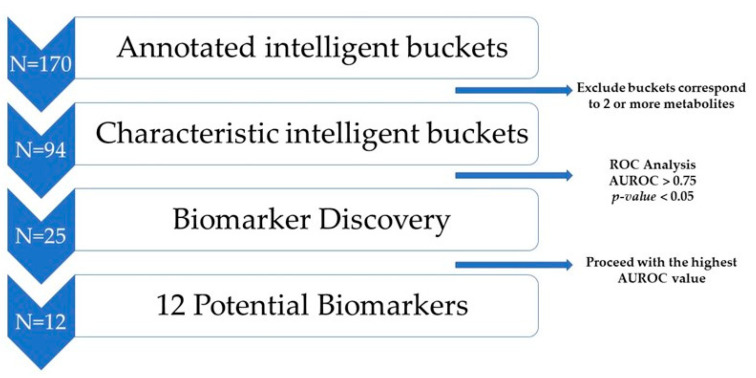
Overview of the buckets’ reduction methodology.

Applying the above criteria, the process concluded with 25 intelligent buckets corresponding to 12 unique metabolites (Supplementary Table S3). Finally, to exclude multiple buckets corresponding to the same metabolite, buckets that demonstrated the higher AUROC (*n* = 12) were kept for further statistical analyses (Supplementary Table S4). The whole procedure is shown in Figure 3.

#### 3.2.3. Discriminant and Pathway Analysis

**Figure 4:**
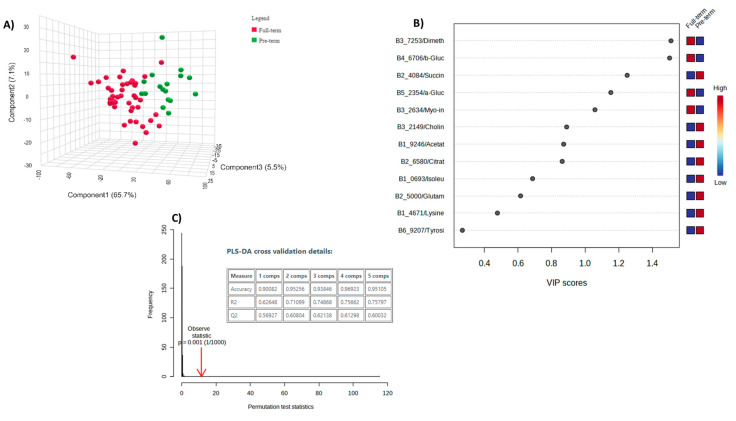
PLS-DA analysis for the AF samples of 60 volunteers. (**A**) Score plot of PLS-DA analysis (R^2^X(cum) = 0.75, Q^2^(cum) = 0.61, accuracy = 0.93, comp No. = 3). The green and red dots correspond to preterm and term classification, respectively; (**B**) VIP plot of the studied metabolites. (**C**) Validation of the PLS-DA analysis, by permutation test statistics, indicates that the extracted model is significantly different from a model built on random data. The permutation tests were carried out with 1000 random permutations, thus providing significance of the model at the 0.001 level.

**Figure 5:**
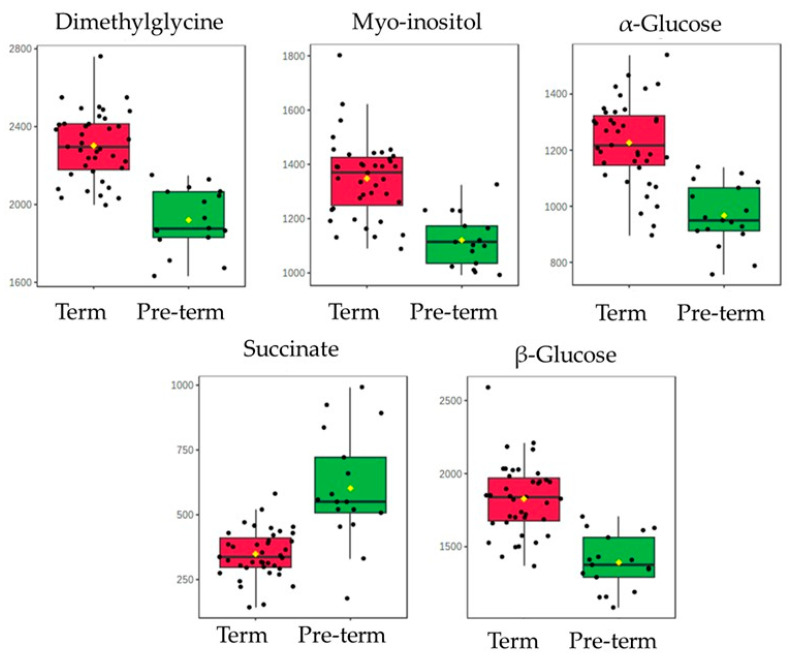
Box plot of the promoted metabolites from the discriminant analysis.

Discriminant analysis was applied to define the metabolites that confirm the discrimination of the two sample categories (full term/preterm). Supervised partial least-squares discriminant analysis (PLS-DA) was employed, using the full-term/preterm classification as the response variable and the 12 potential biomarkers as the independent variables. The extracted 3D score plot manifested the separation of the studied groups across the first component (Figure 4A). The variable importance projection (VIP) plot was then retrieved to identify the variables with the greater discriminative ability (VIP > 1) among the two studied groups. Indicatively, PTB is associated with a lower abundance of dimethylglycine, glucose, and myo-inositol; while in the full-term birth, the production of succinate is fostered (Figure 4B). The model was validated by permutation test statistics (Figure 4C). The box plots of the four promoted metabolites are shown in Figure 5.

Finally, metabolites exhibiting AUROC > 0.75 (Supplementary Table S4) in AF samples were subjected to pathway analysis to relate the promoted metabolic patterns to specific pathways. The results (Supplementary Figure S1) depicted that seven metabolic pathways were significantly enriched (*p* < 0.05) containing at least two compounds, while two of them (alanine, aspartate, and glutamate metabolism and the citrate cycle) had the largest impact (>0.1) (Table 2).

## 4. Discussion

The present study explores the possibility of identifying potential concentration patterns of markers that characterize PTD. Among these 60 asymptomatic women, 43 delivered at term, while 17 delivered prematurely.

Our study supports the hypothesis that PTD is characterized by several changes in the metabolic profile of the fetus as reflected in second-trimester amniotic fluid [10]. The multivariate analysis highlighted that dimethylglycine, glucose, myo-inositol and succinate possessed the highest discriminative ability between the two studied groups. Moreover, pathway analysis revealed the most important metabolic pathways involved in our research hypothesis.

In accordance with prior research results [13], we have observed decreased glucose levels in second-trimester amniotic fluid samples obtained from women that delivered preterm. The decrease in glucose levels may be linked to increased glycolysis, potentially occurring under stressful conditions and reduced utilization of the respiratory chain pathway (as indicated by the rise in succinate levels) partly due to disrupted transplacental flow [15]. Furthermore, a meta-analysis conducted by Liu et al. (2017) supported our observations, indicating that lower glucose levels in early- or mid-trimester AF are associated with PTD [21]. Previous studies have convincingly demonstrated that decreased levels of glucose in AF are linked to the presence of microorganisms in the amniotic cavity and/or an inflammatory response [21,22,23,24]. These findings align with the notion that intraamniotic infection or inflammation (subclinical) plays a causative role in PTD [21].

Our analysis revealed that during the second trimester, women who later experienced PTD exhibited higher levels of succinate in their AF compared to women who carried their pregnancies to term. This discovery aligns with a recent study conducted by Virgiliou et al. in 2017 [14], which observed elevated fumarate levels in mid-trimester AF derived from women who delivered prematurely. Ansari et al. [22] and others [25,26,27] have proposed that succinate and fumarate, vital metabolites in both host and microbial processes, act as potent allosteric inhibitors of 2OG-dependent dioxygenases. Notably, this group of enzymes includes members of the histone demethylase family. Emerging evidence highlights a strong correlation between epigenetic events, particularly histone methylation, T-cell activation, differentiation, and commitment [22,25,26]. Hence, it is plausible that the generation, utilization, and translocation of these metabolites from the mitochondria to the cytosol, facilitating dynamic histone methylation within the nucleus, could potentially contribute to the occurrence of PTD. Given the immunomodulatory effects of succinate, as demonstrated by Al-Mushrif et al. in 2000 albeit in a slightly different setting [23], this finding needs further investigation.

Another noteworthy discovery that aligns with the existing literature [14] pertains to the reduced levels of myo-inositol found in the AF of PTD cases. Myo-inositol is a vital nutrient essential for the growth and viability of human cells. It is worth mentioning that myo-inositol has previously been identified as a significant marker in AF for women experiencing preterm labor without intraamniotic infection or inflammation [14,28,29]. Numerous assumptions may document myo-inositol’s potential role in fetal development and metabolic maturity. Myo-inositol acts as a structural basis for several secondary messengers found in eukaryotic cells, including inositol phosphates, phosphatidylinositol, and phosphatidylinositol phosphate lipids. These secondary messengers are pivotal in various cellular processes, including the regulation of intracellular calcium, gene expression, and lipid metabolism [30]. Current data suggest that a higher content of placental myo-inositol might postpone the initiation of labor by suppressing the synthesis and production of placental eicosanoids. This suppression could result from either a decrease in the availability of arachidonic acid for eicosanoid synthesis or a reduction in the activity of enzymes responsible for eicosanoid production. Moreover, myo-inositol could potentially redirect arachidonic acid metabolism towards generating anti-inflammatory eicosanoids rather than pro-inflammatory ones, thereby regulating the overall equilibrium of pro-inflammatory and anti-inflammatory factors to inhibit the onset of preterm labor. Furthermore, the administration of myo-inositol for the prevention of prematurity has been a subject of study [26].

Dimethylglycine is a derivative of the amino acid glycine. It is important to note that glycine, choline, betaine, and dimethylglycine are interconnected compounds with related metabolic pathways. While a previous metabolomic analysis of healthy pregnant individuals’ AF indicated increased levels of both choline and N-dimethylglycine during the transition from the second to the third trimester [31], there is limited information available regarding dimethylglycine levels in AF among cases of PTD. In the context of our study, dimethylglycine was found to be decreased in AF samples from women who delivered prematurely. Deciphering the role of dimethylglycine in relation to PTD is interesting since current data suggest that dimethylglycine may be engaging in different pathways that influence the regulation of the labor process itself. These effects might be related, at least in part, to its function in bolstering cellular antioxidant activity. It is well-documented that spontaneous PTD is associated with oxidative stress and disturbances in the body’s redox system, often linked with inflammation [14]. Dimethylglycine is involved in multiple metabolic pathways and can facilitate the recycling of other antioxidants, such as glutathione, which is a vital cellular antioxidant [32]. By aiding in the regeneration of other antioxidants, dimethylglycine could indirectly contribute to mitigating oxidative stress and safeguarding cells from damage caused by reactive oxygen species (ROS). Another viable scenario involves its role as a methyl donor. The transfer of a methyl group from betaine to homocysteine, catalyzed by the enzyme betaine-homocysteine methyltransferase, results in the production of methionine and dimethylglycine. Consequently, a deficiency in methyl donors may result in disturbances in metabolism and function [24].

Interestingly, as previously discussed, a recent study [16] found no significant differences in metabolite profiles associated with spontaneous PTD. This outcome may be attributed to variations in stratification and research protocols.

The strengths of this study are evident in its approach to data collection and the rigorous statistical methods applied. The collection of samples during the pre-clinical phase, when women show no symptoms, has the potential to identify individuals at high risk early on. This approach aligns with the findings of Souza et al. in 2019 [4], suggesting that it can help uncover the triggers of preterm labor. Furthermore, a meticulously statistical approach was adopted to thoroughly assess the candidate biomarkers’ diagnostic and prognostic potential. This method yielded a concise set of features that represented the most reliable and noteworthy markers, characterized by AUROC values exceeding 0.75. Regarding limitations, the study’s sample size may pose constraints on the broader applicability of its findings. Additionally, for future investigations, the integration of metabolomic techniques with advanced molecular microbiological methods could offer a deeper exploration of the intricate connections between microbiota and metabolites.

## 5. Conclusions

To conclude, NMR metabolomics’ analysis led to the identification of glucose, succinate, myo-inositol, and dimethylglycine as potential biomarkers of PTD. The results overall support previous data, while they underscore the dynamic nature of AF composition, reflecting the physiological changes occurring throughout pregnancy. The identified specific metabolites and their fluctuations have the potential to serve as biomarkers for monitoring pregnancy progression and identifying aberrations that may require medical attention. Furthermore, our study has highlighted the intricate interplay between maternal and fetal metabolisms within the AF environment. This intergenerational metabolic dialogue can inform our understanding of fetal development and its susceptibility to external factors.

## Figures and Tables

**Table 1:** Sample demographics and pregnancy outcome data by group (*n* = 60).

	Preterm (*n* = 17)	Full Term (*n* = 43)	Mann–Whitney U Test
	% (*n*)	% (*n*)	*p* Value
**Sex**			
Boy	58.8% (10)	51.2% (22)	0.595
Girl	41.2% (7)	48.8% (21)	
**Delivery**			
Cesarean section	17.6% (3)	7% (3)	0.218
Normal delivery	82.4% (14)	93% (40)	
	**Mean (SD)**	**Mean (SD)**	
Gestational age (weeks)	35.3 (2.8)	38.5 (0.9)	<0.001
Amniocentesis (week)	20.31 (2.46)	19.68 (1.82)	0.479
Age (years)	36.54 (2.70)	37.29 (3.63)	0.548
Weight (Kg)	71.82 (10.54)	73.16 (9.11)	0.755
Weight gain (Kg)	12.9 (6.4)	12.9 (5.9)	0.786
Neonatal weight (g)	2695.29 (553.85)	3340.23 (380.77)	<0.001
Neonatal weight (z-scores)	−0.886 (1.06)	0.350 (0.730)	<0.001
Neonatal length (cm)	51.2 (2.2)	51.2 (1.6)	0.744

SD = standard deviation.

**Table 2:** Results of the pathway analysis of the AF samples (in bold, the pathways of importance are depicted).

No.	Pathway	Total	Expected	Hits	Raw *p*	log (*p*)	Holm Adjust	FDR	Impact
1	Aminoacyl-tRNA biosynthesis	48	0.34	4	0.0002	3.6417	0.0192	0.02	0.00
2	Alanine, aspartate, and glutamate metabolism	28	0.20	3	0.0008	3.1015	0.0657	0.03	0.20
3	Glyoxylate and dicarboxylate metabolism	32	0.23	3	0.0012	2.9282	0.0968	0.03	0.03
4	Butanoate metabolism	15	0.11	2	0.0046	2.3397	0.3705	0.10	0.00
5	Citrate cycle (TCA cycle)	20	0.14	2	0.0081	2.0906	0.6494	0.14	0.12
6	Glycolysis/ Gluconeogenesis	26	0.18	2	0.0136	1.8676	1.0000	0.19	0.03
7	Glycine, serine, and threonine metabolism	33	0.23	2	0.0214	1.6686	1.0000	0.26	0.07
8	Phenylalanine, tyrosine, and tryptophan biosynthesis	4	0.03	1	0.0281	1.5511	1.0000	0.30	0.50
9	Nitrogen metabolism	6	0.04	1	0.0419	1.3778	1.0000	0.35	0.00
10	D-Glutamine and D-glutamate metabolism	6	0.04	1	0.0419	1.3778	1.0000	0.35	0.50

## Data Availability

The data presented in the manuscript are available on reasonable request from the corresponding authors. The data are not publicly available due to their containing information that could compromise the privacy of research participants.

## Supplementary Materials

The following supporting information can be downloaded at: https://www.mdpi.com/article/10.3390/metabo13111147/s1, Table S1. Table representing the characteristic chemical shifts of the identified metabolites, Table S2. Table representing the annotated buckets based on the identification procedure. The term “BX_XXXX” corresponds to ppm. Table S3. Illustration of Biomarker Analysis. In total 50 annotated buckets corresponding to unique metabolites, passed the statistical significance analysis (*p* < 0.05). In bold are presented the 25 annotated buckets with AUROC > 0.75 and *p* value < 0.05. Table S4. Buckets corresponding to the higher AUROCs and 12 unique metabolites. Figure S1: Graphical illustration of Pathway Analysis.

## References

[1] Goldenberg R.L., Culhane J.F., Iams J.D., Romero R. (2008). Epidemiology and causes of preterm birth. Lancet.

[2] Vu T.N., Laukens K. (2013). Getting Your Peaks in Line: A Review of Alignment Methods for NMR Spectral Data. Metabolites.

[3] Wojtowicz W., Zabek A., Deja S., Dawiskiba T., Pawelka D., Glod M., Balcerzak W., Mlynarz P. (2017). Serum and urine 1H NMR-based metabolomics in the diagnosis of selected thyroid diseases. Sci. Rep..

[4] Souza R.T., Mayrink J., Leite D.F., Costa M.L., Calderon I.M., Rocha E.A., Vettorazzi J., Feitosa F.E., Cecatti J.G. (2019). Metabolomics applied to maternal and perinatal health: A review of new frontiers with a translation potential. Clinics.

[5] Pang Z., Chong J., Zhou G., de Lima Morais D.A., Chang L., Barrette M., Gauthier C., Jacques P.-É., Li S., Xia J. (2021). MetaboAnalyst 5.0: Narrowing the gap between raw spectra and functional insights. Nucleic Acids Res..

[6] Gil A.M., Duarte D. (2018). Biofluid metabolomics in preterm birth research. Reprod. Sci..

[7] Ansari A., Bose S., You Y., Park S., Kim Y. (2021). Molecular mechanism of microbiota metabolites in preterm birth: Pathological and therapeutic insights. Int. J. Mol. Sci..

[8] Li X., Huang S., Jiao A., Yang X., Yun J., Wang Y., Xue X., Chu Y., Liu F., Liu Y. (2017). Association between ambient fine particulate matter and preterm birth or term low birth weight: An updated systematic review and meta-analysis. Environ. Pollut..

[9] Michaelidou A.-M., Tsakoumaki F., Fotiou M., Kyrkou C., Athanasiadis A.P. (2019). Metabolomics in amniotic fluid. New Technologies and Perinatal Medicine.

[10] Kolvatzis C., Tsakiridis I., Kalogiannidis I.A., Tsakoumaki F., Kyrkou C., Dagklis T., Daniilidis A., Michaelidou A.-M., Athanasiadis A. (2023). Utilizing Amniotic Fluid Metabolomics to Monitor Fetal Well-Being: A Narrative Review of the Literature. Cureus.

[11] Graça G., Diaz S.O., Pinto J., Barros A.S., Duarte I.F., Goodfellow B.J., Galhano E., Pita C., Almeida M.d.C., Carreira I.M. (2012). Can biofluids metabolic profiling help to improve healthcare during pregnancy?. Spectrosc. Int. J..

[12] Graca G., Moreira A.S., Correia A.J.V., Goodfellow B.J., Barros A.S., Duarte I.F., Carreira I.M., Galhano E., Pita C., do Céu Almeida M. (2013). Mid-infrared (MIR) metabolic fingerprinting of amniotic fluid: A possible avenue for early diagnosis of prenatal disorders?. Anal. Chim. Acta.

[13] Graça G., Goodfellow B.J., Barros A.S., Diaz S., Duarte I.F., Spagou K., Veselkov K., Want E.J., Lindon J.C., Carreira I.M. (2012). UPLC-MS metabolic profiling of second trimester amniotic fluid and maternal urine and comparison with NMR spectral profiling for the identification of pregnancy disorder biomarkers. Mol. BioSystems.

[14] Virgiliou C., Gika H.G., Witting M., Bletsou A.A., Athanasiadis A., Zafrakas M., Thomaidis N.S., Raikos N., Makrydimas G., Theodoridis G.A. (2017). Amniotic fluid and maternal serum metabolic signatures in the second trimester associated with preterm delivery. J. Proteome Res..

[15] Graca G., Duarte I.F., Barros A.S., Goodfellow B.J., Diaz S.O., Pinto J., Carreira I.M., Galhano E., Pita C., Gil A.M. (2010). Impact of prenatal disorders on the metabolic profile of second trimester amniotic fluid: A nuclear magnetic resonance metabonomic study. J. Proteome Res..

[16] Hallingström M., Barman M., Savolainen O., Viklund F., Kacerovsky M., Brunius C., Jacobsson B. (2022). Metabolomic profiles of mid-trimester amniotic fluid are not associated with subsequent spontaneous preterm delivery or gestational duration at delivery. J. Matern. -Fetal Neonatal Med..

[17] Giovannopoulou E., Tsakiridis I., Mamopoulos A., Kalogiannidis I., Papoulidis I., Athanasiadis A., Dagklis T. (2022). Invasive prenatal diagnostic testing for aneuploidies in singleton pregnancies: A comparative review of major guidelines. Medicina.

[18] Jacob D., Deborde C., Lefebvre M., Maucourt M., Moing A. (2017). NMRProcFlow: A graphical and interactive tool dedicated to 1D spectra processing for NMR-based metabolomics. Metabolomics.

[19] Xia J., Bjorndahl T.C., Tang P., Wishart D.S. (2008). MetaboMiner–semi-automated identification of metabolites from 2D NMR spectra of complex biofluids. BMC Bioinform..

[20] Murgia F., Monni G., Corda V., Hendren A.J., Paci G., Piras A., Ibba R.M., Atzori L. (2021). Metabolomics analysis of amniotic fluid in euploid foetuses with thickened nuchal translucency by gas chromatography-mass spectrometry. Life.

[21] Gauthier D.W., Meyer W.J., Bieniarz A. (1991). Correlation of amniotic fluid glucose concentration and intraamniotic infection in patients with preterm labor or premature rupture of membranes. Am. J. Obstet. Gynecol..

[22] Romero R., Quintero R., Nores J., Avila C., Hanaoka S., Hagay Z., Merchant L., Hobbins J.C. (1991). Amniotic fluid white blood cell count: A rapid and simple test to diagnose microbial invasion of the amniotic cavity and predict preterm delivery. Am. J. Obstet. Gynecol..

[23] Kirshon B., Rosenfeld B., Mari G., Beifort M. (1991). Amniotic fluid glucose and intraamniotic infection. Am. J. Obstet. Gynecol..

[24] Parthasarathy S., Soundararajan P., Sakthivelu M., Karuppiah K.M., Velusamy P., Gopinath S.C., Pachaiappan R. (2023). The role of prognostic biomarkers and their implications in early detection of preeclampsia: A systematic review. Process Biochem..

[25] Wang M., Wang H., Zheng H., Uhrin D., Dewhurst R.J., Roehe R. (2021). Comparison of HPLC and NMR for quantification of the main volatile fatty acids in rumen digesta. Sci. Rep..

[26] Shyer J.A., Flavell R.A., Bailis W. (2020). Metabolic signaling in T cells. Cell Res..

[27] Wan J., Jiang F., Zhang J., Xu Q., Chen D., Yu B., Mao X., Yu J., Luo Y., He J. (2017). Amniotic fluid metabolomics and biochemistry analysis provides novel insights into the diet-regulated foetal growth in a pig model. Sci. Rep..

[28] Romero R., Mazaki-Tovi S., Vaisbuch E., Kusanovic J.P., Chaiworapongsa T., Gomez R., Nien J.K., Yoon B.H., Mazor M., Luo J. (2010). Metabolomics in premature labor: A novel approach to identify patients at risk for preterm delivery. J. Matern. -Fetal Neonatal Med..

[29] Harman C.R. (2008). Amniotic fluid abnormalities. Semin. Perinatol..

[30] Dessì A., Fanos V. (2013). Myoinositol: A new marker of intrauterine growth restriction?. J. Obstet. Gynaecol..

[31] Orczyk-Pawilowicz M., Jawien E., Deja S., Hirnle L., Zabek A., Mlynarz P. (2016). Metabolomics of human amniotic fluid and maternal plasma during normal pregnancy. PLoS ONE.

[32] Menon R. (2014). Oxidative stress damage as a detrimental factor in preterm birth pathology. Front. Immunol..

