# Supplementary material for: Metabolomic Profiling of Second-Trimester Amniotic Fluid for Predicting Preterm Delivery: Insights from NMR Analysis
Source: Metabolites. 2023 Nov 12;13(11):1147. doi: 10.3390/metabo13111147 (PMC10672859; doi:10.3390/metabo13111147)
Supplement: Supplementary file 1 [file metabolites-13-01147-s001.zip › supplementary.htm]

| **Metabolite name** | **δ (1H NMR chemical shift) ppm** | **Assignment** | **Multiplicity** |
| --- | --- | --- | --- |
| Leucine | 0.96 | (CH_3_)_2_ | t |
| Valine | 1.00, 1.05 | CH_3_, CH_3_ | d, d |
| Isoleucine | 0.96, 1.07 | δCH_3_, βCH_3_ | t, d |
| Lactate | 1.34, 4.13 | βCH_3_, αCH | d, q |
| Alanine | 1.49 | CH_3_ | d |
| Lysine | 1.70-1.90 | δCH_2_, βCH_2_ | m, m |
| Acetate | 1.92 | CH_3_ | s |
| Glutamate | 2.04/ 2.09, 2.38/2.44 | βCH_2_/ β’CH_2_, γCH_2_/ γ’CH_2_ | m, m |
| Acetone | 2.23 | (CH_3_)_2_ | s |
| Acetoacetate | 2.28 | CH_3_ | s |
| Succinate | 2.40 | CH_2_ | s |
| Citrate | 2.53, 2.67 | half CH_2_, half CH_2_ | d, d |
| Dimethylamine | 2.76 | N(CH_3_)_2_ | s |
| Trimethylamine | 2.88 | N(CH_3_)_3_ | s |
| Creatine | 3.01, 3.93 | NCH_3_, NCH_2_ | s, s |
| Choline | 3.21 | N(CH_3_)_3_ | s |
| Myo-inositol | 3.26, 3.53, 3.62 | C5H ring, C1H/C3H ring, C4H/C6H ring | t, dd, dd |
| Betaine | 3.31, 3.92 | N(CH_3_)_3_, CH2 | s, s |
| Glycine | 3.44 | CH_2_ | s |
| Dimethylglycine | 2.90, 3.72 | (CH_3_)_2_, N(CH_2_) | s, s |
| Glucose | 4.66, 5.24, 3.35-4.02 | α-anomeric H, β-anomeric H, various H | d, d, m |
| Threonine | 1.38, 4.27 | γCH_3_, βCH_2_ | d, m |
| Tyrosine | 6.91, 7.20 | C3H ring, C2H ring | d; d |
| Phenylalanine | 7.34, 7.38, 7.43 | C2H/C6H ring, C4H ring, C3H/C5H ring | m, m, m |
| Histidine | 7.10, 8.16 | C4H ring, C2H ring | s, s |
| Formate | 8.5 | CH | s |

Supplementary Material

**Table S1.** Table representing the characteristic chemical shifts of the identified metabolites.

**Table S2.** Table representing the annotated buckets based on the identification procedure. The term “BX_XXXX” corresponds to ppm.

| **No.** | **Bucket** | **Annotation** | **center** | **min** | **max** |
| --- | --- | --- | --- | --- | --- |
| 1 | B0_9566 | Leucine/Isoleucine | 0.956595 | 0.950264 | 0.962926 |
| 2 | B0_9689 | Leucine | 0.968875 | 0.962926 | 0.974825 |
| 3 | B0_9796 | Leucine | 0.979554 | 0.974825 | 0.984283 |
| 4 | B0_9914 | Valine | 0.991377 | 0.984283 | 0.998471 |
| 5 | B1_0083 | Valine | 1.008311 | 0.998471 | 1.01815 |
| 6 | B1_0251 | Isoleucine | 1.025092 | 1.01815 | 1.032033 |
| 7 | B1_0410 | Valine | 1.040957 | 1.032033 | 1.049882 |
| 8 | B1_0571 | Valine | 1.057052 | 1.049882 | 1.064222 |
| 9 | B1_0693 | Isoleucine | 1.069256 | 1.064222 | 1.074291 |
| 10 | B1_0843 | Isoleucine | 1.084283 | 1.077189 | 1.091377 |
| 11 | B1_4554 | Lysine | 1.455449 | 1.450186 | 1.460712 |
| 12 | B1_4671 | Lysine | 1.467119 | 1.460712 | 1.473526 |
| 13 | B1_4810 | Alanine | 1.481002 | 1.473526 | 1.488477 |
| 14 | B1_4976 | Alanine | 1.49763 | 1.488477 | 1.506783 |
| 15 | B1_7094 | Lysine/Leucine | 1.709376 | 1.701291 | 1.717461 |
| 16 | B1_7251 | Lysine/Leucine | 1.725089 | 1.717461 | 1.732717 |
| 17 | B1_7410 | Lysine/Leucine | 1.740955 | 1.732717 | 1.749193 |
| 18 | B1_7574 | Lysine/Leucine | 1.757355 | 1.749193 | 1.765516 |
| 19 | B1_8378 | Lysine | 1.837827 | 1.78184 | 1.893815 |
| 20 | B1_9005 | Lysine | 1.900527 | 1.893815 | 1.90724 |
| 21 | B1_9093 | Lysine | 1.909299 | 1.90724 | 1.911359 |
| 22 | B1_9149 | Lysine | 1.914944 | 1.911359 | 1.918529 |
| 23 | B1_9246 | Acetate | 1.924631 | 1.918529 | 1.930733 |
| 24 | B1_9926 | Glutamate | 1.992594 | 1.9855 | 1.999688 |
| 25 | B2_0066 | Glutamate | 2.006629 | 1.999688 | 2.01357 |
| 26 | B2_0214 | Glutamate | 2.021427 | 2.01357 | 2.029283 |
| 27 | B2_0338 | Glutamate | 2.033784 | 2.029283 | 2.038284 |
| 28 | B2_0459 | Glutamate | 2.045912 | 2.038284 | 2.05354 |
| 29 | B2_0589 | Glutamate | 2.058879 | 2.05354 | 2.064218 |
| 30 | B2_0715 | Glutamate | 2.071465 | 2.064218 | 2.078711 |
| 31 | B2_0863 | Glutamate | 2.086263 | 2.078711 | 2.093814 |
| 32 | B2_1011 | Glutamate | 2.10106 | 2.093814 | 2.108307 |
| 33 | B2_1118 | Glutamate | 2.111816 | 2.108307 | 2.115324 |
| 34 | B2_1196 | Glutamate | 2.119596 | 2.115324 | 2.123867 |
| 35 | B2_2342 | Acetone | 2.234164 | 2.221045 | 2.247284 |
| 36 | B2_2641 | Valine | 2.264141 | 2.259183 | 2.2691 |
| 37 | B2_2720 | Valine | 2.271998 | 2.2691 | 2.274897 |
| 38 | B2_2792 | Valine | 2.279244 | 2.274897 | 2.283592 |
| 39 | B2_2864 | Acetoacetate | 2.286414 | 2.283592 | 2.289237 |
| 40 | B2_2924 | Glutamate | 2.29244 | 2.289237 | 2.295644 |
| 41 | B2_3006 | Glutamate | 2.300602 | 2.295644 | 2.30556 |
| 42 | B2_3127 | Glutamate | 2.31273 | 2.309221 | 2.316239 |
| 43 | B2_3206 | Glutamate | 2.320587 | 2.316239 | 2.324935 |
| 44 | B2_3294 | Glutamate | 2.329435 | 2.324935 | 2.333935 |
| 45 | B2_3407 | Glutamate | 2.340724 | 2.333935 | 2.347513 |
| 46 | B2_3559 | Glutamate | 2.355903 | 2.347513 | 2.364294 |
| 47 | B2_3681 | Glutamate | 2.368108 | 2.364294 | 2.371921 |
| 48 | B2_3755 | Glutamate | 2.375506 | 2.371921 | 2.379091 |
| 49 | B2_3828 | Glutamate | 2.382829 | 2.379091 | 2.386567 |
| 50 | B2_3931 | Glutamate | 2.393127 | 2.386567 | 2.399686 |
| 51 | B2_4084 | Succinate | 2.408382 | 2.399686 | 2.417078 |
| 52 | B2_4570 | Glutamate | 2.456971 | 2.450487 | 2.463454 |
| 53 | B2_4715 | Glutamate | 2.471463 | 2.463454 | 2.479473 |
| 54 | B2_4866 | Glutamate | 2.486643 | 2.479473 | 2.493813 |
| 55 | B2_5000 | Glutamate | 2.499991 | 2.493813 | 2.50617 |
| 56 | B2_5193 | Citrate | 2.519289 | 2.50617 | 2.532409 |
| 57 | B2_5503 | Citrate | 2.550334 | 2.536681 | 2.563988 |
| 58 | B2_6580 | Citrate | 2.657962 | 2.642248 | 2.673675 |
| 59 | B2_6915 | Citrate | 2.691524 | 2.679929 | 2.703118 |
| 60 | B2_7622 | Dimethylamine | 2.762233 | 2.749342 | 2.775124 |
| 61 | B2_8843 | Trimethylamine | 2.884277 | 2.877793 | 2.89076 |
| 62 | B2_9014 | Dimethylglycine | 2.901363 | 2.895184 | 2.907541 |
| 63 | B2_9331 | Tyrosine | 2.933094 | 2.922034 | 2.944154 |
| 64 | B2_9491 | Tyrosine | 2.949112 | 2.944154 | 2.95407 |
| 65 | B3_0133 | Creatine | 3.013262 | 3.00182 | 3.024703 |
| 66 | B3_0340 | Lysine | 3.034009 | 3.028059 | 3.039959 |
| 67 | B3_0435 | Lysine | 3.043544 | 3.039959 | 3.047129 |
| 68 | B3_0528 | Lysine | 3.052773 | 3.047129 | 3.058418 |
| 69 | B3_0637 | Lysine | 3.063681 | 3.058418 | 3.068944 |
| 70 | B3_1162 | Histidine | 3.116236 | 3.10632 | 3.126152 |
| 71 | B3_1378 | Histidine/Phenylalanine | 3.137823 | 3.130424 | 3.145221 |
| 72 | B3_1517 | Histidine/Phenylalanine | 3.151705 | 3.145221 | 3.158189 |
| 73 | B3_1634 | Histidine/Phenylalanine | 3.163376 | 3.158189 | 3.168562 |
| 74 | B3_1732 | Histidine/Phenylalanine | 3.173215 | 3.168562 | 3.177868 |
| 75 | B3_1853 | Histidine | 3.185267 | 3.177868 | 3.192666 |
| 76 | B3_2001 | Histidine | 3.200141 | 3.192666 | 3.207616 |
| 77 | B3_2149 | Choline | 3.214863 | 3.207616 | 3.222109 |
| 78 | B3_2266 | Myo-inositol | 3.226609 | 3.222109 | 3.23111 |
| 79 | B3_2348 | Myo-inositol | 3.234771 | 3.23111 | 3.238432 |
| 80 | B3_2432 | Myo-inositol | 3.243162 | 3.238432 | 3.247891 |
| 81 | B3_2531 | Myo-inositol | 3.253078 | 3.247891 | 3.258265 |
| 82 | B3_2634 | Myo-inositol | 3.263375 | 3.258265 | 3.268486 |
| 83 | B3_2763 | Myo-inositol | 3.276266 | 3.268486 | 3.284046 |
| 84 | B3_2914 | Myo-inositol | 3.291445 | 3.284046 | 3.298844 |
| 85 | B3_3106 | Betaine | 3.310591 | 3.302811 | 3.318371 |
| 86 | B3_3580 | Glucose/Sugar moieties | 3.358035 | 3.350713 | 3.365358 |
| 87 | B3_3692 | Glucose/Sugar moieties | 3.369248 | 3.365358 | 3.373138 |
| 88 | B3_3779 | Glucose/Sugar moieties | 3.377868 | 3.373138 | 3.382597 |
| 89 | B3_3854 | Glucose/Sugar moieties | 3.385419 | 3.382597 | 3.388241 |
| 90 | B3_3930 | Glucose/Sugar moieties | 3.393047 | 3.388241 | 3.397852 |
| 91 | B3_4033 | Glucose/Sugar moieties | 3.403344 | 3.397852 | 3.408836 |
| 92 | B3_4146 | Glucose/Sugar moieties | 3.414633 | 3.408836 | 3.42043 |
| 93 | B3_4240 | Glucose/Sugar moieties | 3.424015 | 3.42043 | 3.4276 |
| 94 | B3_4322 | Glucose/Sugar moieties | 3.432177 | 3.4276 | 3.436754 |
| 95 | B3_4429 | Glycine | 3.442856 | 3.436754 | 3.448958 |
| 96 | B3_4519 | Glucose/Sugar moieties | 3.451933 | 3.448958 | 3.454908 |
| 97 | B3_4592 | Glucose/Sugar moieties | 3.459179 | 3.454908 | 3.463451 |
| 98 | B3_4664 | Glucose/Sugar moieties | 3.466426 | 3.463451 | 3.4694 |
| 99 | B3_4721 | Glucose/Sugar moieties | 3.472146 | 3.4694 | 3.474892 |
| 100 | B3_4789 | Glucose/Sugar moieties | 3.478859 | 3.474892 | 3.482825 |
| 101 | B3_4864 | Glucose/Sugar moieties | 3.48641 | 3.482825 | 3.489995 |
| 102 | B3_4922 | Glucose/Sugar moieties | 3.492207 | 3.489995 | 3.494419 |
| 103 | B3_4975 | Glucose/Sugar moieties | 3.497547 | 3.494419 | 3.500674 |
| 104 | B3_5079 | Glucose/Sugar moieties/Myo-inositol | 3.50792 | 3.500674 | 3.515167 |
| 105 | B3_5222 | Glucose/Sugar moieties/Myo-inositol | 3.522184 | 3.515167 | 3.529202 |
| 106 | B3_5335 | Glucose/Sugar moieties/Myo-inositol | 3.53355 | 3.529202 | 3.537897 |
| 107 | B3_5418 | Glucose/Sugar moieties/Myo-inositol | 3.541788 | 3.537897 | 3.545678 |
| 108 | B3_5481 | Glucose/Sugar moieties/Myo-inositol | 3.548119 | 3.545678 | 3.550559 |
| 109 | B3_5539 | Glucose/Sugar moieties/Valine/Myo-inositol | 3.553916 | 3.550559 | 3.557272 |
| 110 | B3_5615 | Glucose/Sugar moieties/Valine/Myo-inositol | 3.561467 | 3.557272 | 3.565662 |
| 111 | B3_5708 | Glucose/Sugar moieties/Valine/Myo-inositol | 3.570773 | 3.565662 | 3.575884 |
| 112 | B3_5846 | Glucose/Sugar moieties/Myo-inositol | 3.584579 | 3.578782 | 3.590376 |
| 113 | B3_5960 | Glucose/Sugar moieties/Myo-inositol | 3.596021 | 3.590376 | 3.601665 |
| 114 | B3_6059 | Glucose/Sugar moieties/Myo-inositol | 3.605937 | 3.601665 | 3.610208 |
| 115 | B3_6162 | Glucose/Sugar moieties/Myo-inositol | 3.616234 | 3.610208 | 3.62226 |
| 116 | B3_6259 | Glucose/Sugar moieties/Myo-inositol | 3.625922 | 3.62226 | 3.629583 |
| 117 | B3_6335 | Glucose/Sugar moieties/Myo-inositol | 3.633473 | 3.629583 | 3.637363 |
| 118 | B3_6420 | Glucose/Sugar moieties/Isoleucine/Myo-inositol | 3.642016 | 3.637363 | 3.646669 |
| 119 | B3_6554 | Glucose/Sugar moieties/Isoleucine/Myo-inositol | 3.655365 | 3.646669 | 3.66406 |
| 120 | B3_6665 | Glucose/Sugar moieties | 3.666501 | 3.66406 | 3.668942 |
| 121 | B3_6755 | Glucose/Sugar moieties | 3.675502 | 3.668942 | 3.682062 |
| 122 | B3_6889 | Glucose/Sugar moieties | 3.688927 | 3.682062 | 3.695792 |
| 123 | B3_7028 | Glucose/Sugar moieties | 3.702809 | 3.695792 | 3.709827 |
| 124 | B3_7146 | Glucose/Sugar moieties | 3.714632 | 3.709827 | 3.719438 |
| 125 | B3_7253 | Dimethylglycine | 3.725311 | 3.719438 | 3.731184 |
| 126 | B3_7393 | Glucose/Sugar moieties/Glutamate | 3.73927 | 3.731184 | 3.747355 |
| 127 | B3_7548 | Glucose/Sugar moieties/Glutamate | 3.75483 | 3.747355 | 3.762306 |
| 128 | B3_7661 | Glucose/Sugar moieties/Leucine | 3.766119 | 3.762306 | 3.769933 |
| 129 | B3_7728 | Glucose/Sugar moieties/Leucine | 3.772756 | 3.769933 | 3.775578 |
| 130 | B3_7799 | Glucose/Sugar moieties/Alanine/Lysine | 3.779926 | 3.775578 | 3.784273 |
| 131 | B3_7866 | Glucose/Sugar moieties/Alanine/Lysine | 3.786638 | 3.784273 | 3.789003 |
| 132 | B3_7937 | Glucose/Sugar moieties/Alanine/Lysine | 3.793732 | 3.789003 | 3.798461 |
| 133 | B3_8018 | Glucose/Sugar moieties/Alanine/Lysine | 3.801817 | 3.798461 | 3.805173 |
| 134 | B3_8076 | Glucose/Sugar moieties/Alanine/Lysine | 3.807614 | 3.805173 | 3.810055 |
| 135 | B3_8149 | Glucose/Sugar moieties | 3.814937 | 3.810055 | 3.819819 |
| 136 | B3_8231 | Glucose/Sugar moieties | 3.823099 | 3.819819 | 3.826379 |
| 137 | B3_8340 | Glucose/Sugar moieties | 3.834006 | 3.826379 | 3.841634 |
| 138 | B3_8466 | Glucose/Sugar moieties | 3.846592 | 3.841634 | 3.85155 |
| 139 | B3_8605 | Glucose/Sugar moieties | 3.860474 | 3.85155 | 3.869399 |
| 140 | B3_8755 | Glucose/Sugar moieties | 3.875501 | 3.869399 | 3.881603 |
| 141 | B3_8939 | Glucose/Sugar moieties | 3.893884 | 3.881603 | 3.906165 |
| 142 | B3_9110 | Glucose/Sugar moieties | 3.911046 | 3.906165 | 3.915928 |
| 143 | B3_9229 | Betaine/Sugar moieties | 3.922946 | 3.915928 | 3.929963 |
| 144 | B3_9368 | Creatine/Sugar moieties | 3.936752 | 3.929963 | 3.943541 |
| 145 | B3_9513 | Glucose/Sugar moieties/Histidine | 3.951321 | 3.943541 | 3.959101 |
| 146 | B3_9646 | Glucose/Sugar moieties/Histidine | 3.964593 | 3.959101 | 3.970085 |
| 147 | B3_9768 | Glucose/Sugar moieties/Histidine | 3.976797 | 3.970085 | 3.98351 |
| 148 | B3_9872 | Glucose/Sugar moieties/Histidine | 3.987247 | 3.98351 | 3.990985 |
| 149 | B3_9933 | Glucose/Sugar moieties/Histidine | 3.99335 | 3.990985 | 3.995714 |
| 150 | B3_9985 | Glucose/Sugar moieties/Histidine | 3.99846 | 3.995714 | 4.001206 |
| 151 | B4_0039 | Glucose/Sugar moieties/Histidine/Phenylalanine | 4.003876 | 4.001206 | 4.006546 |
| 152 | B4_0091 | Glucose/Sugar moieties/Histidine/Phenylalanine | 4.009139 | 4.006546 | 4.011733 |
| 153 | B4_0149 | Glucose/Sugar moieties/Histidine/Phenylalanine | 4.01486 | 4.011733 | 4.017987 |
| 154 | B4_0240 | Glucose/Sugar moieties/Histidine/Phenylalanine | 4.024013 | 4.017987 | 4.030039 |
| 155 | B4_6457 | b-Glucose | 4.645674 | 4.632173 | 4.659175 |
| 156 | B4_6706 | b-Glucose | 4.670617 | 4.659175 | 4.682058 |
| 157 | B5_2354 | a-Glucose | 5.235374 | 5.224695 | 5.246053 |
| 158 | B5_2549 | a-Glucose | 5.254901 | 5.246053 | 5.263749 |
| 159 | B6_9044 | Tyrosine | 6.9044 | 6.898374 | 6.910426 |
| 160 | B6_9207 | Tyrosine | 6.920723 | 6.914545 | 6.926901 |
| 161 | B7_1029 | Histidine | 7.102873 | 7.086016 | 7.119731 |
| 162 | B7_1911 | Tyrosine | 7.19105 | 7.1806 | 7.2015 |
| 163 | B7_2150 | Tyrosine | 7.215001 | 7.205314 | 7.224688 |
| 164 | B7_3301 | Phenylalanine | 7.330104 | 7.323086 | 7.337121 |
| 165 | B7_3499 | Phenylalanine | 7.34986 | 7.337121 | 7.362598 |
| 166 | B7_3840 | Phenylalanine | 7.383956 | 7.378769 | 7.389142 |
| 167 | B7_4225 | Phenylalanine | 7.422476 | 7.413399 | 7.431553 |
| 168 | B7_4394 | Phenylalanine | 7.439409 | 7.431553 | 7.447266 |
| 169 | B8_1620 | Histidine | 8.161985 | 7.875182 | 8.448788 |
| 170 | B8_5068 | Formate | 8.506759 | 8.448788 | 8.564729 |

**Table S3.** Illustration of Biomarker Analysis. In total 50 annotated buckets corresponding to unique metabolites, passed the statistical significance analysis (*p < 0.05*). In bold are presented the 25 annotated buckets with AUROC > 0.75 and *p value < 0.05*. The term “BX_XXXX” corresponds to ppm.

| **No.** | **Annotated bucket** | **AUC** | ***p* value** |
| --- | --- | --- | --- |
| **1** | **B3_7253/Dimethylglycine** | **0.94721** | **2.90 × 10^−10^** |
| **2** | **B4_6706/b-Glucose** | **0.92911** | **2.57 × 10^−8^** |
| **3** | **B3_2634/Myo-inositol** | **0.917044** | **2.17 × 10^−7^** |
| **4** | **B5_2354/a-Glucose** | **0.914027** | **4.26 × 10^−8^** |
| **5** | **B1_0693/Isoleucine** | **0.907994** | **2.85 × 10^−7^** |
| **6** | **B4_6457/b-Glucose** | **0.907994** | **4.32 × 10^−8^** |
| **7** | **B3_2432/Myo-inositol** | **0.901961** | **2.27 × 10^−7^** |
| **8** | **B5_2549/a-Glucose** | **0.900452** | **9.13 × 10^−7^** |
| **9** | **B1_0843/Isoleucine** | **0.892911** | **6.08 × 10^−7^** |
| **10** | **B2_4084/Succinate** | **0.889894** | **7.75 × 10^−8^** |
| **11** | **B2_5000/Glutamate** | **0.868778** | **1.31 × 10^−6^** |
| **12** | **B3_2531/Myo-inositol** | **0.868778** | **2.33 × 10^−5^** |
| **13** | **B3_0528/Lysine** | **0.84917** | **1.98 × 10^−6^** |
| **14** | **B3_0435/Lysine** | **0.831071** | **2.53 × 10^−6^** |
| **15** | **B3_2149/Choline** | **0.825038** | **3.91 × 10^−6^** |
| **16** | **B3_0340/Lysine** | **0.812971** | **0.000395** |
| **17** | **B1_4671/Lysine** | **0.809955** | **0.000269** |
| **18** | **B2_4866/Glutamate** | **0.809955** | **5.20 × 10^−5^** |
| **19** | **B2_3931/Glutamate** | **0.797888** | **0.000135** |
| **20** | **B2_6580/Citrate** | **0.79638** | **6.36 × 10^−5^** |
| **21** | **B1_4554/Lysine** | **0.788839** | **0.001145** |
| **22** | **B1_9246/Acetate** | **0.779789** | **0.001182** |
| **23** | **B2_4715/Glutamate** | **0.772247** | **0.00055** |
| **24** | **B6_9207/Tyrosine** | **0.758673** | **0.000842** |
| **25** | **B2_0066/Glutamate** | **0.75264** | **0.001453** |
| 26 | B2_5193/Citrate | 0.748115 | 0.002489 |
| 27 | B1_9926/Glutamate | 0.745098 | 0.007291 |
| 28 | B1_4976/Alanine | 0.73454 | 0.005749 |
| 29 | B2_5503/Citrate | 0.733032 | 0.000824 |
| 30 | B2_4570/Glutamate | 0.730015 | 0.002283 |
| 31 | B2_6915/Citrate | 0.723982 | 0.001645 |
| 32 | B1_4810/Alanine | 0.722474 | 0.010009 |
| 33 | B2_9491/Tyrosine | 0.721719 | 0.00505 |
| 34 | B1_9005/Lysine | 0.708899 | 0.016799 |
| 35 | B1_9149/Lysine | 0.707391 | 0.030287 |
| 36 | B3_4429/Glycine | 0.702866 | 0.010537 |
| 37 | B3_2914/Myo-inositol | 0.699849 | 0.002437 |
| 38 | B2_3828/Glutamate | 0.698341 | 0.005984 |
| 39 | B2_9014/Dimethylglycine | 0.698341 | 0.02667 |
| 40 | B2_1196/Glutamate | 0.693816 | 0.02596 |
| 41 | B2_3755/Glutamate | 0.689291 | 0.019109 |
| 42 | B3_0133/Creatine | 0.687783 | 0.040387 |
| 43 | B3_2348/Myo-inositol | 0.680241 | 0.009912 |
| 44 | B1_0571/Valine | 0.675716 | 0.042023 |
| 45 | B2_3681/Glutamate | 0.6727 | 0.018286 |
| 46 | B6_9044/Tyrosine | 0.6727 | 0.006139 |
| 47 | B2_2641/Valine | 0.660633 | 0.026263 |
| 48 | B2_3294/Glutamate | 0.644042 | 0.048307 |
| 49 | B2_8843/Trimethylamine | 0.639517 | 0.019054 |
| 50 | B3_2763/Myo-inositol | 0.612368 | 0.081803 |

**Table S4.** Buckets corresponding to the higher AUROCs and 12 unique metabolites. The term “BX_XXXX” corresponds to ppm.

| **No.** | **Annotated bucket** | **AUC** | ***p* value** |
| --- | --- | --- | --- |
| 1 | B3_7253/Dimethylglycine | 0.94721 | 2.90 × 10^−10^ |
| 2 | B4_6706/b-Glucose | 0.92911 | 2.57 × 10^−8^ |
| 3 | B3_2634/Myo-inositol | 0.917044 | 2.17 × 10^−7^ |
| 4 | B5_2354/a-Glucose | 0.914027 | 4.26 × 10^−8^ |
| 5 | B1_0693/Isoleucine | 0.907994 | 2.85× 10^−7^ |
| 6 | B2_4084/Succinate | 0.889894 | 7.75 × 10^−8^ |
| 7 | B2_5000/Glutamate | 0.868778 | 1.31 × 10^−6^ |
| 8 | B3_0528/Lysine | 0.84917 | 1.98× 10^−6^ |
| 9 | B3_2149/Choline | 0.825038 | 3.91 × 10^−6^ |
| 10 | B2_6580/Citrate | 0.79638 | 6.36 × 10^−5^ |
| 11 | B1_9246/Acetate | 0.779789 | 0.001182 |
| 12 | B6_9207/Tyrosine | 0.758673 | 0.000842 |


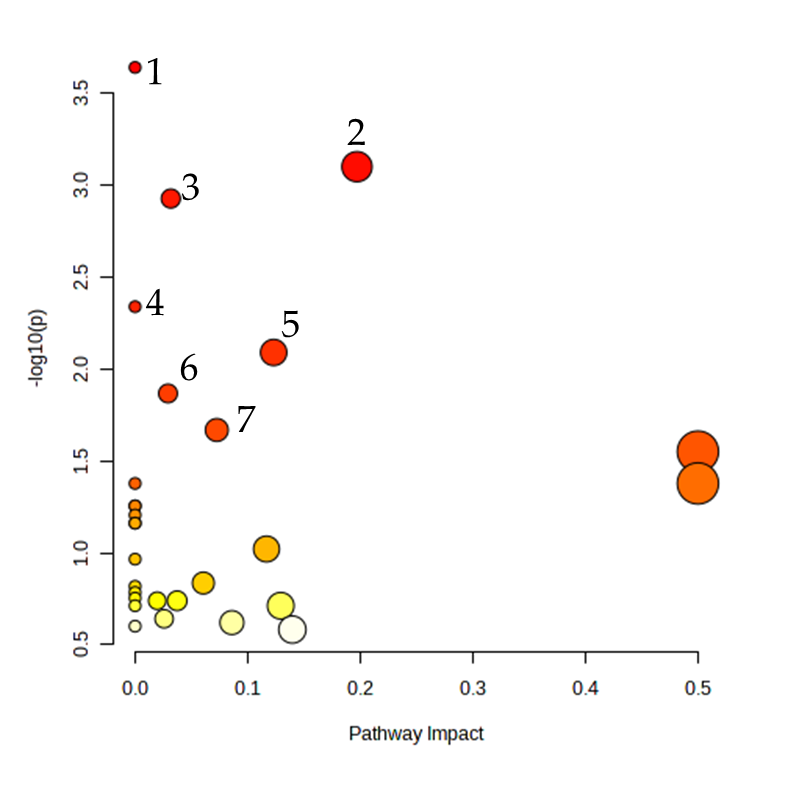


**Figure S1.** Graphical illustration of Pathway Analysis. The numbers correspond to the Table XX results.
